# Star-Shaped Magnetic-Plasmonic Au@Fe_3_O_4_ Nano-Heterostructures for Photothermal Therapy

**DOI:** 10.1021/acsami.2c04865

**Published:** 2022-06-16

**Authors:** Beatrice Muzzi, Martin Albino, Alessio Gabbani, Alexander Omelyanchik, Elena Kozenkova, Michele Petrecca, Claudia Innocenti, Elena Balica, Alessandro Lavacchi, Francesca Scavone, Cecilia Anceschi, Gaia Petrucci, Alfonso Ibarra, Anna Laurenzana, Francesco Pineider, Valeria Rodionova, Claudio Sangregorio

**Affiliations:** † Institute of Chemistry of Organometallic Compounds − C.N.R., 50019 Sesto Fiorentino (FI), Italy; ‡ Department of Biotechnology, Chemistry and Pharmacy, University of Siena 1240, I-53100 Siena, Italy; § Department of Chemistry ‘Ugo Schiff’ & INSTM, University of Florence, 50019 Sesto Fiorentino (FI), Italy; ∥ Institute of Physics, Mathematics and Information Technology, Immanuel Kant Baltic Federal University, 236008 Kaliningrad, Russia; ⊥ Department of Chemistry and Industrial Chemistry & INSTM, University of Pisa, 56126 Pisa, Italy; # Laboratorio de Microscopias Avanzadas (LMA), Universidad de Zaragoza, 50018 Zaragoza, Spain; ∇ Department of Experimental and Clinical Biomedical Sciences, University of Florence, 50134 Firenze, Italy

**Keywords:** core@shell, heterostructures, Au@Fe_3_O_4_, nanostar, magnetic-plasmonic, photothermal therapy

## Abstract

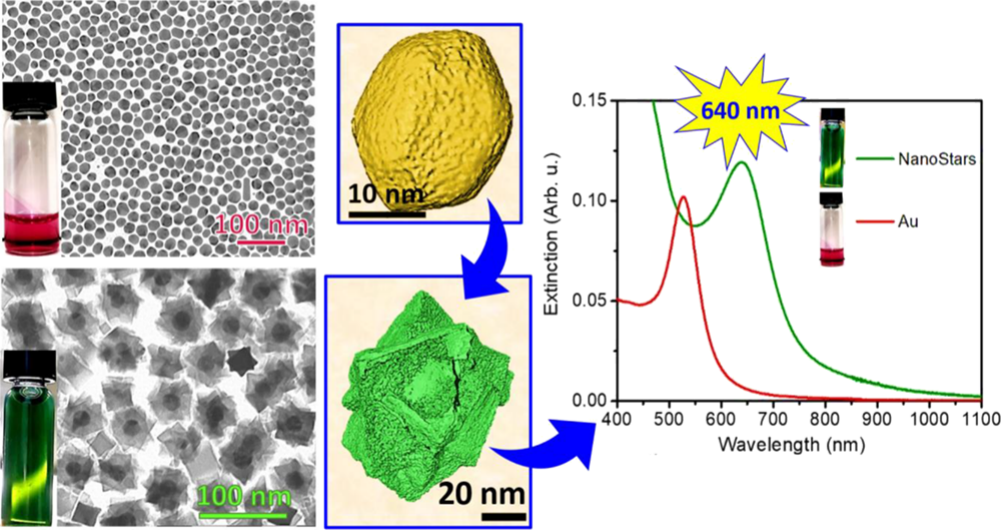

Here, we synthesize a Au@Fe_3_O_4_ core@shell system with a highly uniform unprecedented star-like shell morphology with combined plasmonic and magnetic properties. An advanced electron microscopy characterization allows assessing the multifaceted nature of the Au core and its role in the growth of the peculiar epitaxial star-like shell with excellent crystallinity and homogeneity. Magnetometry and magneto-optical spectroscopy revealed a pure magnetite shell, with a superior saturation magnetization compared to similar Au@Fe_3_O_4_ heterostructures reported in the literature, which is ascribed to the star-like morphology, as well as to the large thickness of the shell. Of note, Au@Fe_3_O_4_ nanostar-loaded cancer cells displayed magneto-mechanical stress under a low frequency external alternating magnetic field (few tens of Hz). On the other hand, such a uniform, homogeneous, and thick magnetite shell enables the shift of the plasmonic resonance of the Au core to 640 nm, which is the largest red shift achievable in Au@Fe_3_O_4_ homogeneous core@shell systems, prompting application in photothermal therapy and optical imaging in the first biologically transparent window. Preliminary experiments performing irradiation of a stable water suspension of the nanostar and Au@Fe_3_O_4_-loaded cancer cell culture suspension at 658 nm confirmed their optical response and their suitability for photothermal therapy. The outstanding features of the prepared system can be thus potentially exploited as a multifunctional platform for magnetic-plasmonic applications.

## Introduction

Multifunctional hybrid nanoparticles (NPs) have attracted enormous interest for the possibility of combining two or more functionalities from different inorganic materials in the same nanostructure. Within this framework, magnetic-plasmonic NPs (MP-NPs) are characterized by a unique combination of magnetic and optical properties,^1−3^ which can provide a multifunctional theranostic platform for biomedicine.^4−8^ On the one hand, the magnetic properties enable the possibility of remotely controlling the movement of such nanostructures by the application of an external field, opening the way for magnetic targeting and drug delivery nanosystems. Moreover, the application of an alternating magnetic field (AMF) at different frequencies can produce local therapeutic heating by magnetic fluid hyperthermia (high frequency application) or cell damage by magneto-mechanic stress (low frequencies application).^9^ Finally, magnetic resonance imaging can be performed using the NPs as the contrast agent.^10−12^ On the other hand, the noble metal moiety provides an intense optical response ascribed to the plasmonic resonance, which empowers optical imaging and can act as a nanosource of heat for thermal therapy triggered remotely by photon irradiation.^13−16^ Interestingly, the coupling of a noble metal with a metal oxide shell can induce a red shift of the plasmon peak toward the near-infrared (NIR) spectral region,^17,18^ which is a more attractive spectral range for biomedical application due to the optical transparency of biological tissues between 600 and 1300 nm.^19−21^ Magnetic and plasmonic features can also be applied synergistically: the heating induced by the simultaneous application of an AMF and laser irradiation can be combined, potentially increasing the efficiency of the magneto-photothermal treatments.^2,22^ Besides biomedicine, MP-NPs have a great potential in technological applications such as information encryption, magnetic field sensing, and mechanochromic devices owing to magnetically dependent plasmonic excitation in the anisotropic particles.^3,23,24^

Due to their high biocompatibility, magnetite (Fe_3_O_4_) and maghemite (γ-Fe_2_O_3_) as magnetic components and Au as the plasmonic one are to date the most promising and studied materials in MP-NPs.^1,25,12^ The physical properties of the heterostructure can be tuned by modifying the morphology of the two components and the way they are coupled. Accordingly, in the recent past, Au/Fe_3_O_4_ MP-NPs have been synthesized in many different architectures: Fe_3_O_4_@Au core@shell (CS),^8,3^ flower-like with a ferrite core,^26,27^ dumbbell-like dimers,^6−8,22,27,28^ and Janus nanostars.^2^ Nevertheless, obtaining Au NPs uniformly covered with a highly ordered, thick Fe_3_O_4_ shell (at least 15–20 nm) is still a challenging task.^22,31,32^ A homogeneous and thick iron oxide coating of the gold core would enable a more precise control of the plasmonic resonance energy, inducing a more significant red shift compared to a dumbbell or flower-like structure^27^ with an incomplete covering of the Au surface. Indeed, the localized surface plasmon resonance (LSPR) frequency strongly depends on the local permittivity which surrounds the Au surface and thus on the shell permittivity and thickness.^29,30^ On the other hand, the achievement of a magnetically ordered thick spinel ferrite shell is a fundamental requirement (high magnetic moment) for the profitable implementation of the heterostructures in most of the above-mentioned applications.

Even though the epitaxial growth of Fe_3_O_4_ on the Au substrate has been largely investigated,^31,32^ the process is still not completely rationalized, due to the several complex interactions involved, that is, weak adhesion energy or crystallographic lattice mismatch.^33,34^ Switzer et al. investigated the deposition of a Fe_3_O_4_ film by molecular beam epitaxy (MBE), pulsed laser deposition, and laser ablation on different faces of gold^31^ and found that Fe_3_O_4_ grows with different orientations depending on the gold exposed face.^31,32^ Moreover, the synthetic method adopted for the Fe_3_O_4_ shell formation plays a role in the shell growth process.^6,27,35^ In this respect, the seed-mediated growth through thermal decomposition of the iron precursor has been established as one of the most promising approaches to obtain highly crystalline NPs with a precise control on the size and shape.

Here, we report the synthesis of a CS Au@Fe_3_O_4_ MP-heterostructures with an unprecedented star-like morphology of average size of ca. 60 nm, comprising an Au core and a highly uniform Fe_3_O_4_ shell. The Au@Fe_3_O_4_ nanostars were synthesized by seeded-growth approach through thermal decomposition of metal precursors and were successfully transferred to water solution. The extended and advanced electron microscopy characterization of the crystallographic structure and chemical composition analysis revealed the presence of a 20 nm multifaceted gold core homogeneously surrounded by an anisotropic magnetite shell (20 nm). A plasmonic resonance shifted toward NIR spectral region (640 nm) was observed for the nanostars, which fits the first biologically transparent window, prompting their application in photothermal treatment and optical imaging. Remarkably, we reached the largest plasmonic resonance wavelength achievable in Au@Fe_3_O_4_ CS systems, as confirmed by analytical calculations. On the other hand, an in-depth magnetic and magneto-optical characterization highlighted the high magnetic moment of the Au@Fe_3_O_4_ MP-NPs and confirmed the purity of the magnetite phase of the nanostar shell.

Before assessing the biomedical application of this multifunctional platform, we demonstrated the Au@Fe_3_O_4_ nanostar biocompatibility on endothelial colony forming cells (ECFCs), a subtype of endothelial progenitor cells. We then proved the photothermal effects after 658 nm light exposure and the nano-shell induction of magneto-mechanical stress by an external AMF on cell viability of MCF7, a breast cancer cell line.

## Experimental Section

### Chemicals and Materials

Gold(III) chloride trihydrate (HAuCl_4_·3H_2_O, 99.8% from Strem Chemicals Inc.), iron(III) acetylacetonate (Fe(acac)_3_, 99% from Sigma-Aldrich), oleic acid (OA, 90%, Aldrich), oleylamine (Ol-NH2, 98%, Aldrich), polyethyleneimine branched (PEI, average MW ∼ 25,000 from Sigma-Aldrich), benzyl ether (Bz_2_O, 98%, Aldrich), dimethyl sulfoxide anhydrous (DMSO, ≥99.9%, Aldrich), ethanol (EtOH, 99.8%, Fluka), and toluene (99%, Aldrich) were used without any further purification.

### Synthesis of Au Seeds

Gold nanocrystals (NCs) were prepared by reduction of HauCl_4_·3H_2_O (120 mg, 0.03 mmol) by oleylamine (3 mL, 9.12 mmol) in benzyl ether (20 mL). The mixture was heated to 120 °C at 4 °C/min under N_2_ flux with vigorous stirring and kept at this temperature for 45 min. Once the mixture was cooled to room temperature, the obtained Au NCs were precipitated by EtOH and successive centrifugation. This washing procedure was repeated several times. The obtained product was dispersed in toluene, giving a stable colloid suspension.

### Synthesis of Au@Fe_3_O_4_

A mixture of Fe(acac)_3_ (0.177 g, 0.5 mmol), Au NCs (0.011 g in ca. 5 mL of toluene), oleylamine (0.53 g, 2 mmol), and oleic acid (0.565 g, 2 mmol) in 50 mL of benzyl ether was heated up to 220 °C at 5 °C/min under N_2_ flow and kept at this temperature for 2 h. Then, the reaction was heated up to reflux (∼300 °C) at 6 °C/min and kept at this temperature for 2 h before cooling it down to room temperature. The MP-NPs were precipitated with a permanent magnet after addition of EtOH to the suspension and washed several times. Afterward, Au@Fe_3_O_4_ were coated with PEI and stored in water.

### Phase Transfer by Ligand-Exchange with PEI

Au@Fe_3_O_4_ (20 mg) were dispersed in toluene (20 mL), added to a solution of PEI (20 mg) in DMSO (5 mL), sonicated for 1 h, and finally incubated at room temperature for 12 h in a rotating agitator. The precipitate was magnetically separated with a permanent magnet, washed several times first with DMSO and then with ethanol, and finally re-dispersed in milliQ water (20 mL), giving a stable colloid suspension.

### Characterization Techniques

Transmission electron microscopy (TEM, CM12 PHILIPS equipped with a LaB_6_ filament operating at 100 kV) was employed to determine morphology and size distribution of MP-NPs. The mean diameter and the size distribution of each sample were obtained by statistical analysis over more than 100 NPs, using the Image Pro-Plus software. Powder X-ray diffraction (XRD) data were recorded using a Bruker New D8 ADVANCE ECO diffractometer equipped with a Cu Kα (1.5406 Å) radiation source and operating in θ–θ Bragg–Brentano geometry at 40 kV and 40 mA. The measurements were carried out in the range 25–70°, with a step size of 0.03° and collection time of 1 s. Elemental analysis was performed in triplicate by a Varian 720-ES inductively coupled plasma–atomic emission spectrometer (ICP-AES). Scanning transmission electron microscopy (STEM) images were acquired by a probe-corrected Titan, working at 300 kV. The microscope is equipped with a high-angle annular dark-field (HAADF) detector (Fischione) and an EDAX detector for energy-dispersive X-ray (EDX) spectroscopy measurements. In order to obtain the crystalline structure of the different phases present in the system, an image-corrected Titan (300 kV) high-resolution electron transmission microscopy (HRTEM) was used. Tomography was carried out with FEI tomography acquisition software at a Tecnai F30, while tomogram reconstruction was carried out by Inspect 3D and Amira 3D reconstruction software after the acquisition of 140 images. Scanning electron microscopy (SEM) images were acquired by a FIB/SEM TESCAN GAIA 32016 equipped with an EDAX Octane Elect Super detector. The microscope hosts a 30 kV Triglav electron column and a Cobra Focused Ion Beam column with a monoisotopic^69^ Ga source.

Magnetic properties were investigated using a Quantum Design MPMS SQUID magnetometer on randomly oriented pressed powder pellets. The field was always applied parallel to the pellet plane. The zero field cooled/field cooled (ZFC/FC) procedure was performed applying a 5 mT probe field.

Optical extinction spectra were recorded on a commercial spectrophotometer (Jasco V-670) in the 300–2200 nm range. Magnetic circular dichroism (MCD) spectra were recorded at room temperature and under ±1.4 Tesla of applied field within the same spectral range using an in-house built setup, which modulates the polarization of light between left-handed and right-handed using a photo-elastic modulator and takes advantage of phase sensitive detection with a lock-in amplifier (for further information, see ref (30)). Extinction and MCD spectra were recorded on a polystyrene (PS) film of the MP-NPs. The nanocomposite film was prepared by mixing 50 μL of a toluene dispersion of the NPs with 100 μL of a 10% w/w PS solution in toluene. The dispersion was drop-cast on a microscopy cover glass, and after solvent evaporation, a thin film was obtained.

Photothermal experiments were performed exposing 40 mL of *a* ≈ 0.8 mg/mL water dispersion of Au@Fe_3_O_4_ nanostars (optical density of ≈1.6 in a 3 mm quartz capillar) to a laser diode (THORLABS), while monitoring the temperature with a fiber-optic sensor (Fotemp), immersed in the solution but carefully kept far from the light illumination path. Two different laser diodes were used for the experiments, one at 658 nm, with a power density of 300 mW/cm^2^, and one at 785 nm, with a power density of 1000 mW/cm^2^.

### Cytotoxicity Assay and Cellular Uptake Study

ECFCs (EGM-2 culture medium; Lonza) were seeded in six-well plates at a density of 1.5 × 10^5^ cells per well in a humidified atmosphere with 5% CO_2_ and then incubated with a culture medium (2 mL per well) containing Au@Fe_3_O_4_ nanostars at increasing concentrations 10, 20, and 50 μg/mL for 24 h. Cell cytotoxicity was determined with trypan blue staining: 20 μL of cell suspensions was resuspended with an equal volume of 0.4% (w/v) trypan blue solution prepared in 0.81% NaCl and 0.06% (w/v) dibasic potassium phosphate. Viable and non-viable cells (trypan blue positive) were counted separately using a dual-chamber hemocytometer and a light microscope.

Magneto-mechanical stress and optical properties were assessed on the breast cancer cell line (MCF7). MCF7 cells were grown in culture dishes 35 mm diameter as a monolayer in DMEM supplemented with 10% FBS. Cells were incubated with Au@Fe_3_O_4_ nanostars (50 μg/mL) for 24 h. Then, cells treated either with Au@Fe_3_O_4_ nanostars or vehicle (H_2_O_2_) were exposed for 30 min to external AMF oscillating at low frequencies (10 Hz with 160 mT amplitude). After that, the exposure cell suspensions were re-seeded, placed again in a humidified atmosphere with 5% CO_2_ for additionally 24 h, and counted the next day with trypan blue staining.

To test the photothermal effect after light exposure, vehicle- and Au@Fe_3_O_4_ nanostar-treated MCF7 were washed two times with phosphate buffered saline (Invitrogen) and detached with trypsin. After centrifugation, the pellets were resuspended in 15 μL of DMEM and placed with a capillary tip in a quartz capillary which was then subjected to 658 nm laser for 30 min. Cell suspensions were re-seeded and counted the next day with trypan blue staining.

Optical microscopy was used to assess qualitative intracellular uptake of Au@Fe_3_O_4_ nanostars. Cellular images were acquired through the EVOS xl core microscope (AMG, Advanced Microscopy Group). For statistical analysis, the data were analyzed using GraphPad Prism6 and Origin and expressed as a mean value ± SD. Statistical analysis was performed using one-way ANOVA.

## Results and Discussion

**Figure 1:**
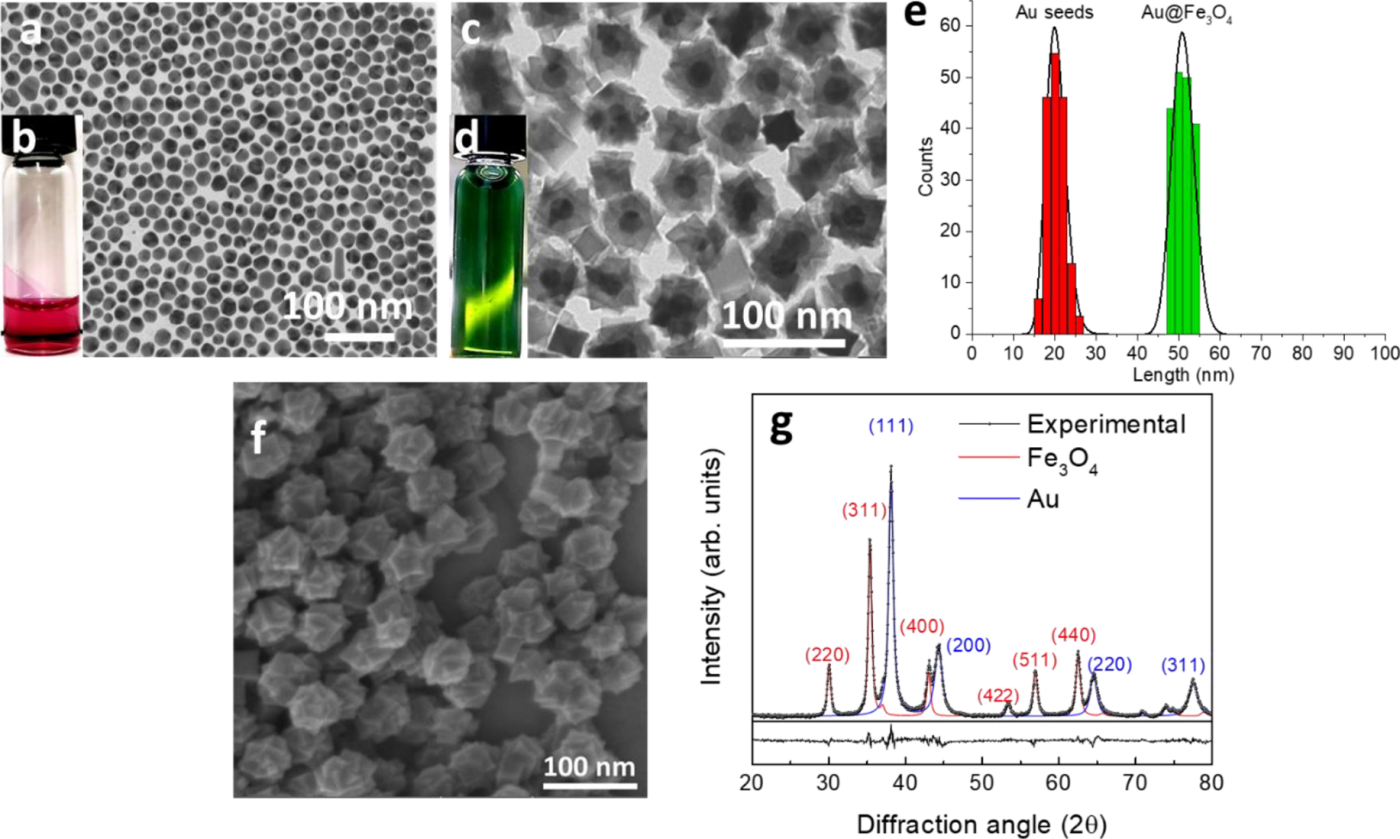
Au@Fe_3_O_4_ nanostars: (a,b) TEM images of Au seeds (ca. 20 nm) and picture of a suspension of the same NPs in toluene; (c,d) TEM micrograph of Au@Fe_3_O_4_ nanostars with the corresponding image of the same MP-NPs dispersed in water; (e) size distribution of the Au seed and Au@Fe_3_O_4_ nanostars; (f) SEM image of the Au@Fe_3_O_4_ nanostars; (g) multiphase Rietveld fitting of the experimental powder X-ray diffraction pattern (dots-black line); the difference between calculated and experimental intensities is also shown.

Au@Fe_3_O_4_ nanostars were prepared through thermal decomposition of iron acetylacetonate in the presence of oleylamine-capped Au NCs, according to a modified seeded-growth approach outlined by Fantechi et al.^27^ Oleylamine was chosen as a surfactant due to the weak coordination at the gold surface, which leads to the formation of a dynamic layer/barrier on NPs (absorption and desorption equilibrium)^36^ allowing not only control of the growth of the NPs by itself but also the nucleation of another inorganic phase onto their surface.^27^ To ensure colloidal stability in water (see Figure 1d), the obtained nanostars were coated with biocompatible amino-rich cationic polymer, PEI.^37,38^ The preliminary morphological and statistical analysis by TEM of Au^(0)^ seeds (Figure 1a,e) shows faceted NPs of 20 nm with a rather sharpsize distribution (±5 nm). In Figure 1c, a representative image of the obtained Au@Fe_3_O_4_ nanostars (average size of 60 ± 10 nm) is displayed, with the corresponding size distribution (Figure 1e). The image reveals the presence of a higher electron density region in the core, attributed to the Au^(0)^, surrounded by a brighter anisotropic shell of iron oxide with several sharp tips (average size of 20 ± 2 nm). Moreover, SEM imaging confirms that the star-like 3D morphology is shared by the almost totality of the NPs in the sample, which appears as an assembly of star-shaped NPs of comparable size (Figure 1f).

The XRD pattern (Figure 1g) of the nanostars shows all the main peaks characteristic of the cubic spinel structure of Fe_3_O_4_ (red pattern, space group *Fd*3̅*m*), while the main reflexes at 38.2° and 44.4° are ascribed to the diffraction planes (111) and (200) of the fcc Au^(0)^ (blue pattern, space group *Fm*3̅*m*). The Rietveld analysis of the pattern displays for the *Fd*3̅*m* structure of Fe_3_O_4_ an average crystallite size of 20(1) nm with lattice parameter *a* = 0.8394(2) nm, and for the gold seeds crystallized in the face-centered cubic *Fm*3̅*m* structure, an average crystallite size of 12(1) nm, *a* = 0.4081(2) nm. The size of gold crystals is smaller than the value obtained from the statistical analysis of TEM micrographs (20 nm), pointing out that the nature of the seeds is two single cubic crystals interpenetrated. However, the obtained lattice parameter is coherent with the literature.^39^ On the contrary, the crystallite size of Fe_3_O_4_ component is similar to the average size obtained from TEM analysis, indicating a high crystallinity level of the shell.

**Figure 2:**
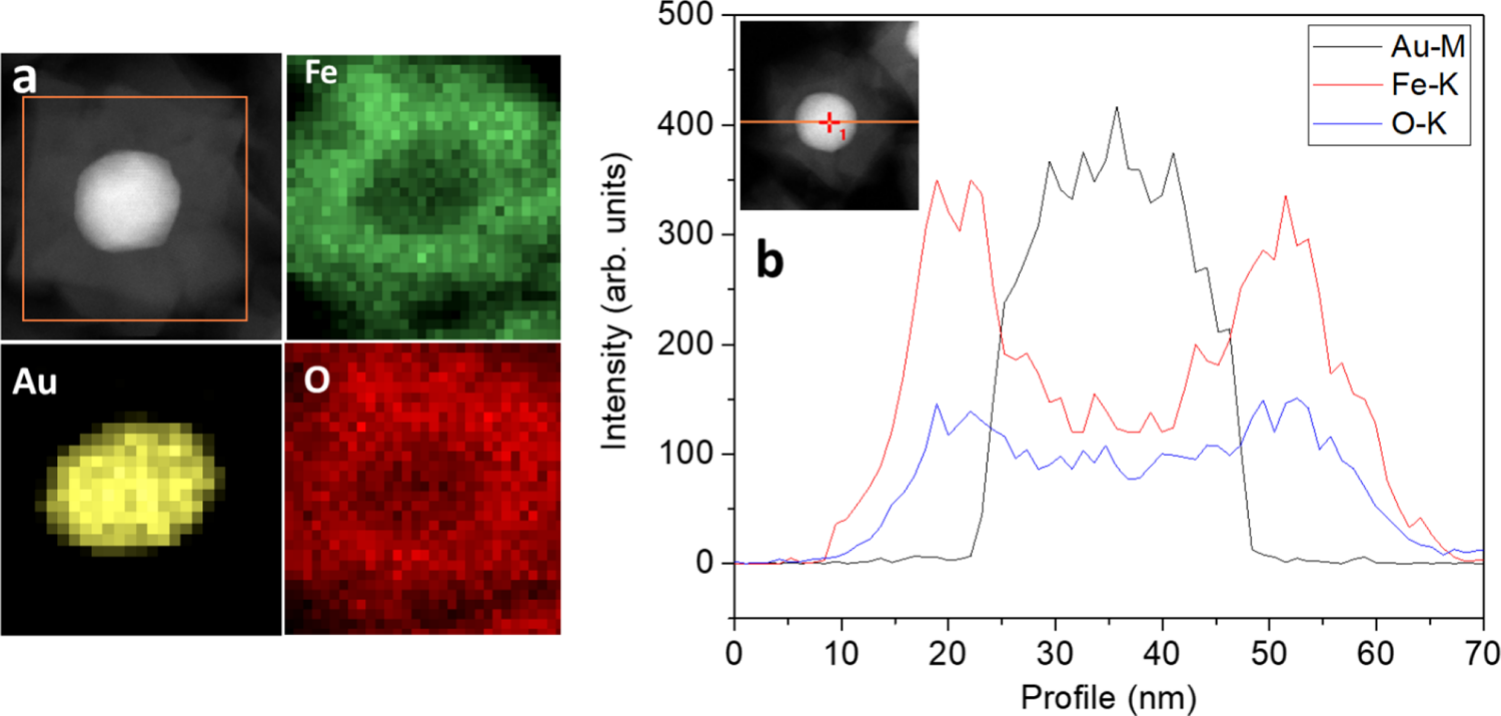
(a) STEM-HAADF image of a single Au@Fe_3_O_4_ NP where the brightest part corresponds to the Au^(0)^ core, while the darker one to the multidomain magnetite shell and STEM-EDX mapping for the chemical composition analysis of the lattice; (b) EDX profiling analysis of a single CS nanostar, along the orange line in the STEM-HAADF image in the inset: black, red and blue lines correspond to Au–M, Fe–K, and O–K radiative relaxations, respectively.

To perform highly resolved mapping and profiling measurements for chemical composition analysis of Au@Fe_3_O_4_, images in STEM-HAADF mode and STEM-EDX analysis were recorded. In Figure 2a, the HAADF image of a single Au@Fe_3_O_4_ nanostars is shown. This analysis confirms the results reported above, displaying the brighter high electron density core region, attributed to Au^(0)^, and the low electron density shell ascribed to the iron oxide. The well-defined core-shell architecture of the nanostars is also clearly displayed by STEM-EDX element mapping distribution, showing the presence of gold in a 22(2) nm core region alone, while iron and oxygen are distributed in the shell (Figure 2a). Since the images were acquired in transmission mode, these two elements were recognized also in the core region, confirming that Au is completely encapsulated by the magnetite shell (Figure 2b).

**Figure 3:**
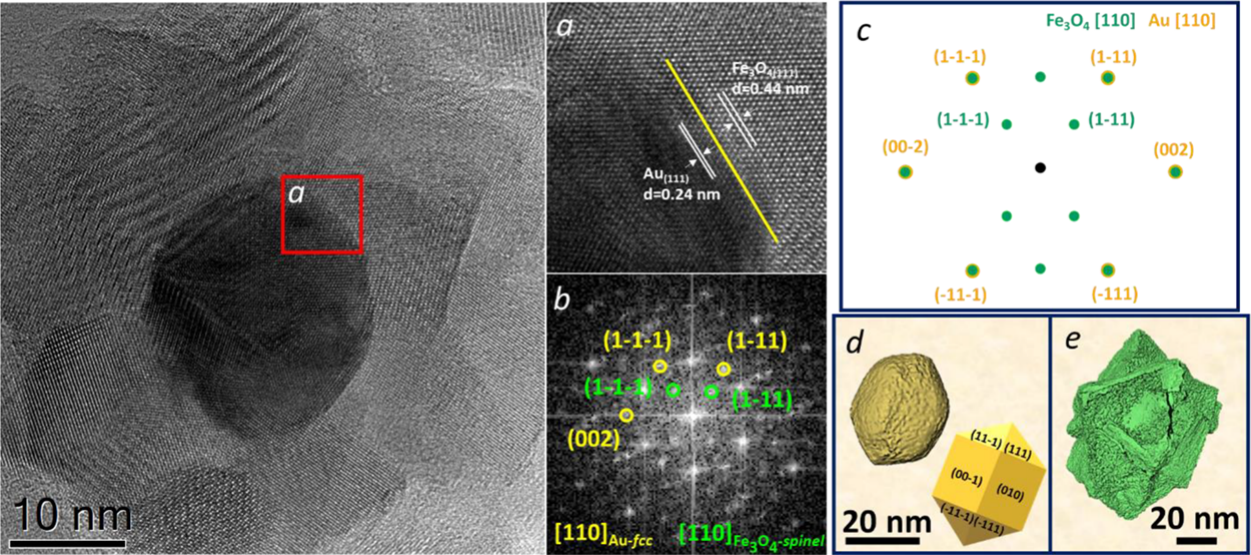
Left: HRTEM image of the Au@Fe_3_O_4_ nanostars; right: (a,b) magnification of the interface region selected in the red square a with the fringe patterns related to Au and Fe_3_O_4_, and its FFT analysis; (c) schematic representation of the reciprocal space diffractogram; (d,e) tomography models of the Au^(0)^ seed with its 3D graphical description with crystallographic faces identified, and Au@Fe_3_O_4_ nanostars, respectively (for details, see Supporting Information).

With the aim to understand the crystalline structure of Au@Fe_3_O_4_ heterostructures at the sub-nanometric level, HRTEM images were acquired, and their local fast Fourier transformation (FFT) patterns were analyzed. In Figure 3 (left panel), the image of an NP is shown as a representative of the whole sample, displaying two regions with different morphologies related to the shell and to the core. To clarify the gold@magnetite epitaxial relationship, the interface region (red square) was taken into account and the corresponding magnification is shown in Figure 3a. Its FFT analysis confirms the presence of the cubic spinel structure of Fe_3_O_4_ in the shell and of the fcc structure of Au^(0)^ in the core, highlighting the crystallographic planes involved in the epitaxial growth (Figure 3b). As reported by the model (Figure 3c), a schematic representation of the reciprocal space diffractogram (Figure 3b) reveals an epitaxial relationship between Au[110](1 −1 −1)//Fe_3_O_4_[110](1 −1 −1) planes. This indicates that the interface, marked by the yellow line, is parallel to the (1 −1 1) Au plane and (1 −1 1) Fe_3_O_4_ plane and that the Fe_3_O_4_ unit cell is matched by the double of the Au one. Thus, the shell’s growth along the <111> directions leads to the formation of several semi-octahedrons of Fe_3_O_4_, which correspond to the star’s spikes. Coherent interfaces are formed when the lattice misfit is ≤10%.^40,41^ However, when nanoscale materials are considered, lattice plastic deformation and surface strain can lead to even larger mismatch. In our case, the lattice misfit, estimated using the formula (d_002_Fe_3_O_4_ – d_001_Au_bulk_)/d_001_Au_bulk_ and relying on lattice site coincidence relationship is 3.3%, in good agreement with the theoretical one (3%) calculated for an ideal bulk Fe_3_O_4_ grown on an Au substrate.^31^ Switzer et al.^32^ studied the growth of Fe_3_O_4_ film by electrodeposition on Au substrates exposing different surfaces, (110), (100), or (111). They demonstrated that Fe_3_O_4_ grows along preferential directions, depending on the face exposed by the single crystal of the noble metal.^32^ On the other hand, for a polycrystalline Au substrate, no preferential orientation is observed. Thus, in our case, we can argue that our nobel metal seeds present a geometry with different faces exposed.^42^ This geometry was crucial to obtain a complete coating. This is shown in Figure 3d where the Au seed modeling, obtained by tomography analysis, displays different crystallographic faces at the surface. This configuration helps the homogeneous Fe_3_O_4_ growth, avoiding the dimer or flower-like structures, which conversely are formed when single faced seeds are used, and the Fe_3_O_4_ growth occurs preferentially only along one or two directions.^28^ Therefore, the different directions for the epitaxial growth of Fe_3_O_4_ up to a 20 nm thickness led to a final star-like shape of the magnetite shell, as it is modelized after tomography analysis (Figure 3e, for more details see Supporting Information) and as it is shown by SEM images.

**Figure 4:**
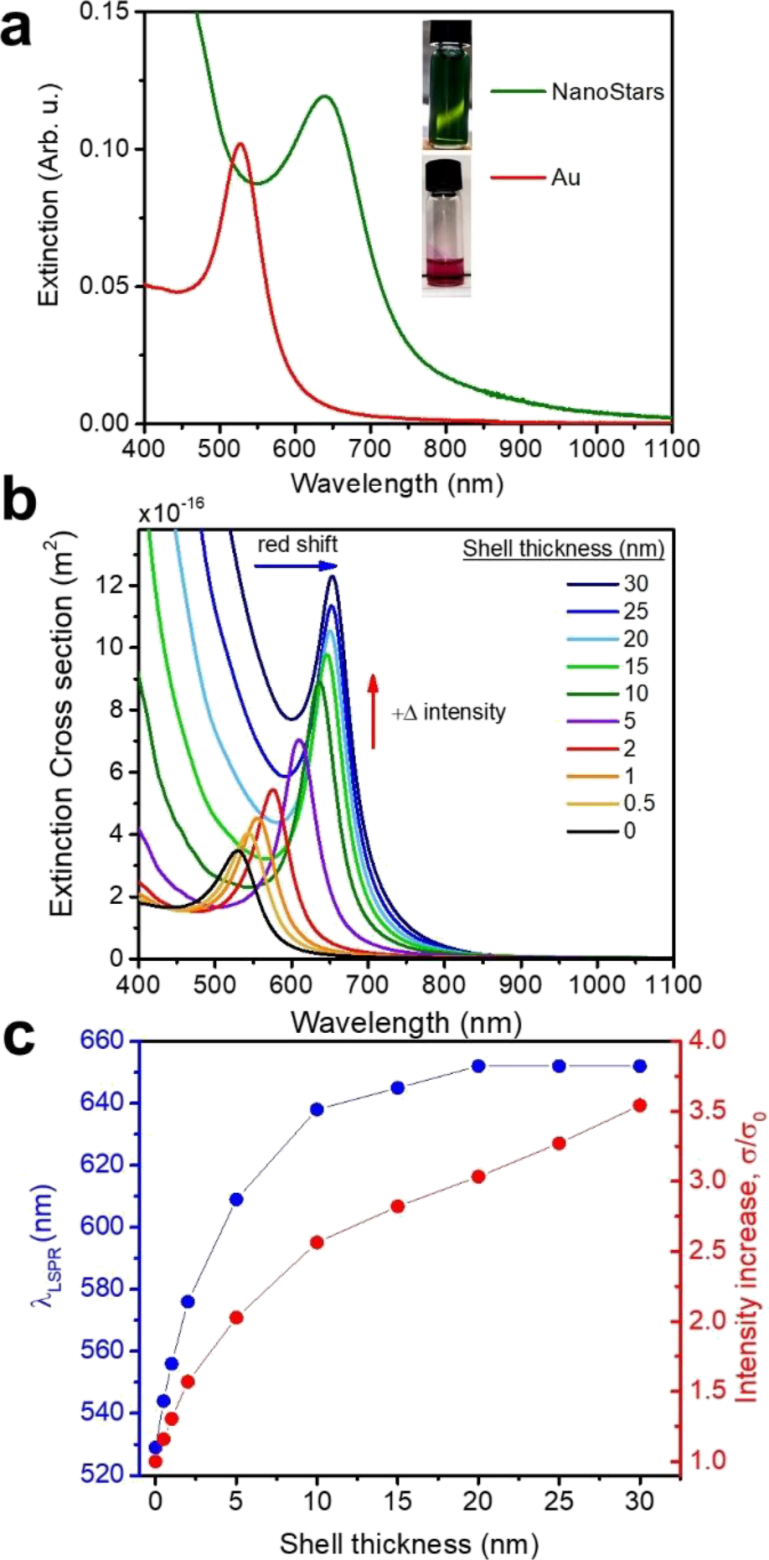
(a) Extinction spectra of Au NPs (red line) in toluene and Au@Fe_3_O_4_ nanostars (green line) dispersed in water, and images of the Au seeds and Au@Fe_3_O_4_ nanostars dispersions; (b) extinction cross section calculated analytically for a CS NP constituted of a 20 nm Au core and shell of different thickness (from 0 to 30 nm); the blue and red arrows indicate the red shift and the increase in intensity, respectively, as a consequence of the increase in shell thickness; (c) localized surface plasmon resonance (LSPR) wavelength (blue curve, left axis) and relative change in cross section intensity at the LSPR maximum (red curve, right axis) as a function of the shell thickness.

In Figure 4a, the experimental extinction spectrum of the AuNPs reveals a plasmonic resonance at 530 nm (red line^43^), which red-shifts at 640 nm (green line) when the gold seeds are surrounded by the magnetite shell. This behavior is ascribed to the higher dielectric permittivity of the oxide shell with respect to the solvent.^27,28,44^ The optical cross section per particle of the nanostars at 640 nm is 6.4 × 10^–16^ m^2^, calculated from the optical spectrum considering the Au weight fraction determined by ICP-AES and the core diameter obtained by TEM analysis. The obtained cross section is larger than the one calculated for pure Au NPs (3.5 × 10^–16^ m^2^),^45^ which demonstrates low optical losses due to the iron oxide shell, potentially ensuring an efficient interaction of the NPs with light in plasmon-based applications. To rationalize the effect of the shell permittivity on the optical response in Au@Fe_3_O_4_ CS systems, we performed analytical calculations of the extinction cross section (s) employing the quasi-static approximation of Mie theory for CS NPs (more details are provided in Supporting Information). The calculations (Figure 4b) were performed for a 20 nm Au core and shells of various thicknesses (from 0 to 30 nm), using the experimental dielectric functions of Au and ferrite iron oxide.^46,47^ An increase in intensity and a red shift of the plasmonic resonance peak with the increase of the shell thickness is observed in the calculations, ascribed to the increase in the dielectric function that surrounds the plasmonic core. However, it clearly emerges that such changes in the plasmonic resonance wavelength reach a saturation at a shell thickness of about 15–20 nm, after which a further increase of the shell size has negligible effects on the position of the plasmonic peak (Figure 4c), consistent with the fact that the local electric field starts to decay stepping away from the Au surface. The intensity of the cross section at the plasmonic peak further increases for thickness above 20 nm, due to the increased contribution of the optical transitions of the shell, which start to be significant and comparable to the contribution coming from the plasmonic core. However, for thickness larger than 20 nm, the iron oxide optical transitions introduce losses, partially masking the plasmonic resonance peak (Figures S2 and S3) and are thus undesirable for plasmon-based applications. We can thus conclude that for the Au@Fe_3_O_4_ CS architecture, 20 nm of shell thickness is an optimal size in order to achieve a significantly red-shifted plasmonic resonance without adding to much optical losses due to the Fe_3_O_4_ transitions. The comparison between the calculations and the experimental spectra reveals a good agreement considering the average shell thickness measured by TEM analysis (≈20 nm). Remarkably, we reached the maximum red shift achievable in Au@Fe_3_O_4_ homogeneous CS systems. Even if a more pronounced red shift can be achieved in shape anisotropic Au nanostructures, such as nanorods, these structures are known to be thermodynamically unstable, rearranging to spheres upon heating.^48^ As a consequence, shifting the resonance while maintaining a thermally stable spherical Au core is a good strategy to avoid thermal reshaping and modification of the optical response upon heating in photothermal therapy. Moreover, a more pronounced shift of the plasmonic resonance toward the infrared is reached in our nanostars with respect to other Au@Fe_3_O_4_ CS NPs reported in the literature, where typically plasmonic resonances below 600 nm are reported.^27,28,49^ Such increased red shift was achieved due to an homogeneous and thick (>15 nm) coating of the Au core with the Fe_3_O_4_ shell, as pointed out by our calculations. This result empowers the exploitation of these CS nanostars in photothermic treatments and optical imaging, as the plasmonic resonance fits the first transparent window (600–1300 nm), where light has its maximum penetration depth in biological tissues.^50−53^

**Figure 5:**
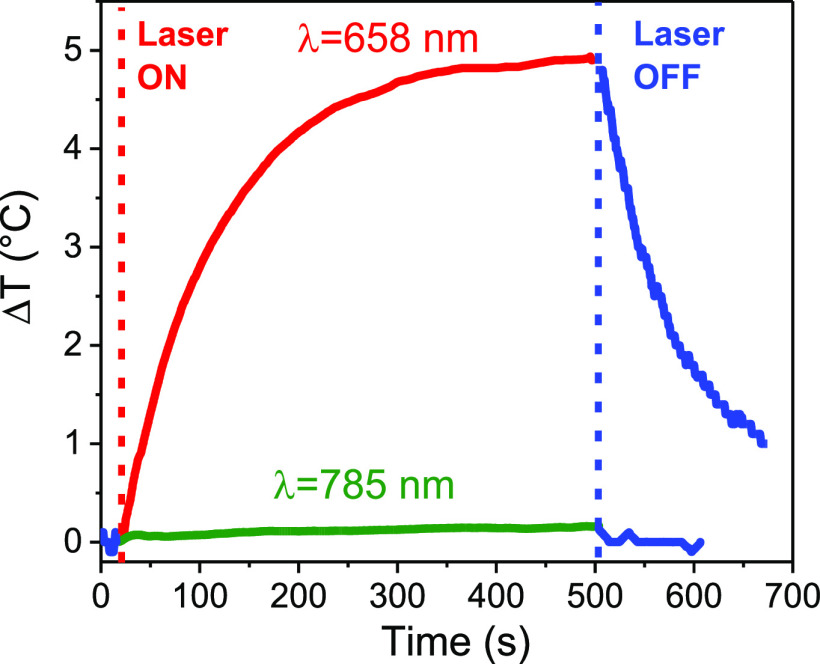
Time-dependent temperature build-up in PEI-coated water dispersion of Au@Fe_3_O_4_ nanostars, irradiated by a laser diode at 658 nm (red curve, laser power 300 mW/cm^2^) and with a laser diode at 785 nm (green curve, laser power 1000 mW/cm^2^). Data points corrected after switching off the light source are marked in blue.

The possibility of using these nanostructures for photothermal treatments was demonstrated by a proof-of-concept photothermal experiment. A water dispersion of the Au@Fe_3_O_4_ NPs (0.8 mg/mL, with an optical density of 1.6 in a 3 mm quartz tube) was irradiated with a laser diode at wavelength close to the plasmonic resonance (λ = 658 nm; laser power 300 mW/cm^2^), and the temperature increase of the solution (Figure 5) was monitored during the irradiation time. The temperature increase of the measurement system without the nanostars (a 3 mm quartz capillary filled with the same volume of water) was subtracted from the one of the sample. A maximum temperature increase of 5 °C was reached after 8 min of illumination at 658 nm, with an initial temperature increase of 0.05 °C/s, while the temperature rapidly falls after switching off the irradiation source. The maximum temperature reached is comparable with previous experiments carried out in the literature with a similar irradiation power on Au nanostructures,^14,54^ and a larger temperature increase is potentially achievable using a laser diode at higher power density (generally up to hundreds of Watts is achievable by commercial laser diodes). On the other hand, irradiating the sample far from the plasmonic resonance maximum, with a 785 nm laser diode (1000 mW/cm^2^) caused negligible temperature increase (0.2 °C), pointing out that the photothermal properties are triggered by the excitation of the plasmonic resonance in the nanostars. Remarkably, the photothermal response exhibits an excellent reproducibility, as confirmed by measuring the temperature increase during 5 cycles of ON/OFF switching of the 658 nm laser diode (see Figure S7), also demonstrating excellent thermal stability of the plasmonic properties.

**Figure 6:**
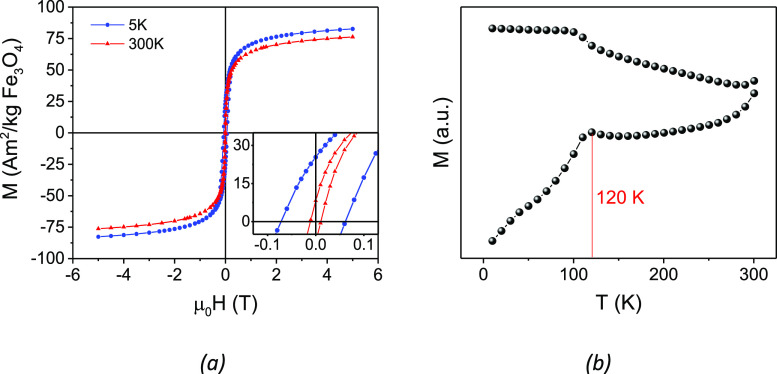
(a) Hysteresis loops of Au@Fe_3_O_4_ measured at 300 K (red) and 5 K (blue); the magnification of the low field region is shown in the inset; (b) temperature dependence of the magnetization recorded after ZFC and FC procedures, applying a constant field of 5 mT.

Magnetic measurements indicate that our system is characterized by M_S_ values, estimated from the high field data, of 76 and 83 A m^2^ kg^–1^, at room and low temperature, respectively (Figure 6a). Data are normalized to the magnetite content as determined by ICP analysis (Au 23% w/w, Fe 55% w/w). The observed M_S_ and M_R_ values are close to those of bulk magnetite^55^ and considerably larger than those reported in the literature for Au-Fe_3_O_4_ heterostructures^56^ or irregularly shaped (octapod) iron oxide NPs of similar size.^57,58^ This result highlights how the epitaxial growth on multifaceted Au seeds favors the formation of a highly ordered magnetic spin system. Indeed, the formation of well-defined, sharp facets, leads to a lower surface disorder in the NC corresponding to a decreased effective surface anisotropy, which in turns favors spin alignment.^59,60^

The Au@Fe_3_O_4_ nanostars display magnetic irreversibility both at room and low temperature with coercive fields of 11 and of 72 mT, respectively, and reduced remnant magnetization (*M*_R_) 11% at 300 K and 30% at 5 K (Figure 6a, inset). However, despite the highly anisotropic shape, the effective magnetic anisotropy estimated from the temperature dependence of the coercive force (see Supporting Information) is only 5.15 ×10^4^ J/m^3^, which is, however, in the upper limit of the range typically observed for bulk magnetite (1–5 ×10^4^ J/m^3^).^61^ This result suggests that the large anisotropies previously reported for magnetite octapods are rather dictated by spin disorder associated to the growth process of the spinel phase,^57,58^ than to an intrinsic shape effect arising from the high degree of spin canting at corners and edges.

The ZFC and FC curves (Figure 6b) displayed magnetic irreversibility up to the highest investigated temperature (300 K), typical of nanosized magnetic materials with blocking temperature larger than room temperature. The kink at ca. 120 K is attributed to the Verwey transition,^62^ which is a fingerprint of the magnetite iron oxide phase.

To further confirm the electronic structure and the oxidation state of the iron ferrite nanostructures, MCD measurements were performed. In iron oxide structures, the analysis of the MCD spectrum allows for the discrimination between maghemite and magnetite, which is quite challenging for NPs, as they tend to oxidize in air, a process which is faster for small NPs (below 10 nm).^21,63^ For this investigation, the nanostars were dispersed in a polymer film, revealing no significant change in the plasmonic optical response with respect to the solution measurement (Figure S4).

**Figure 7:**
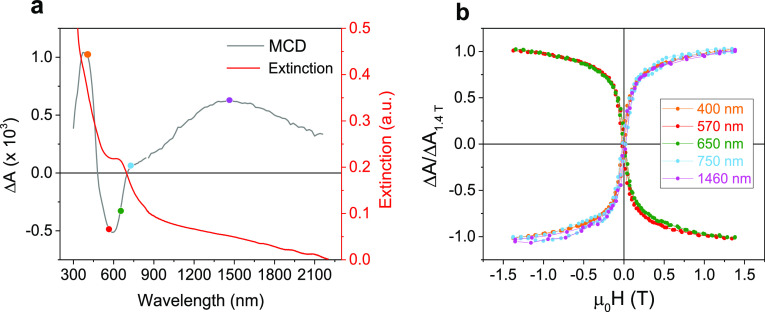
(a) Room temperature MCD spectrum of Au@Fe_3_O_4_ nanostars embedded in a polymer film collected at 1.4 Tesla (gray line) and corresponding extinction spectrum (red line); (b) normalized MCD hysteresis loops collected at different wavelengths, highlighted as colored dots in panel (a).

The MCD spectrum of the sample, collected at 1.4 Tesla, is reported in Figure 7a, together with its extinction spectrum. In magnetic-plasmonic heterostructures, depending on the relative amount of the plasmonic and the magnetic phases, the spectral contribution of both can be detected. A typical weak derivative-like line shape is expected for the LSPR in the MCD spectrum, arising from the magnetic field induced splitting between the circular magnetoplasmonic modes excited by right (left) circularly polarized light.^63−67^ However, since the MCD signal is proportional to the magnetization of the sample, the spectrum is here dominated by the magneto-optical (MO) transitions of the iron oxide phase.^68^ This is also confirmed by simulating the MCD contribution expected for the LSPR starting from the absorption peak parameters and using the cyclotron shift approximation, which yields a differential absorption (Δ*A*) value in the order of 10^–5^, two orders of magnitude smaller than the experimental spectrum (for details, see Supporting Information and Figure S5). According to these considerations, we can conclude that with this architecture and this combination of materials, an eventual interaction between the plasmonic and the magnetic part is not detectable with MCD. Different from what has been reported for Fe_3_O_4_@Au^69^ or Ag@FeCo^70^ CS NPs and plasmonic-single molecule magnet^71^ hybrid nanoarchitectures, no surface-enhanced magneto-optical effects are observed in the system presented here.

The MCD spectrum shows several features, whose full interpretation is not straightforward, as many broad overlapping transitions are present. Referring to the work by Fontijn et al.,^20^ the optically detectable electronic transitions arising from spinel ferrites materials can be classified as crystal field (CF) and charge transfer (CT) transitions. CF transitions occur between the *3*d states of Fe cations, whose degeneracy is lifted by the ligand field created by the fcc lattice of the oxygen anions, while CT transitions occur either with the electron transfer between neighboring cations [intervalence CT (IVCT)] or between cations in different crystallographic sites [intersublattice CT (ISCT)]. The positive peak in the region 300–500 nm in our MCD spectrum is the convolution of several MO signals of magnetite due to ISCT and IVCT transitions (more detailed discussion is reported in the Supporting Information), while the positive peak at 1450 nm is ascribed to IVCT between Fe^3+^ and Fe^2+^ in octahedral sites.^20^ Moreover, a broad negative peak at 590 nm is observed, which is typically present in magnetite and absent in maghemite, ascribed to the transition [Fe^2+^]*t*_2g_ → [Fe^2+^]*e*_g_.^21^ This analysis points toward a magnetite phase of the iron oxide, due to the presence of the MO signals associated with transitions involving Fe^2+^ ions, which are not present in maghemite.

To further confirm the presence of a homogeneous magnetic phase, hysteresis loops at different wavelengths were collected (Figure 7b). Notice that different from magnetometric hysteresis loops, the sign of the MCD hysteresis loops follows the sign of the spectrum at the corresponding wavelength, hence the presence of positive and negative loops in Figure 7b. All the hysteresis loops acquired show the same field dependence, confirming that only one magnetic phase is present in the heterostructures, namely, magnetite. The exclusive presence of the magnetite phase is consistent with the large size (>15 nm) of the iron oxide domains, where the ratio of surface atoms is small, thus limiting surface oxidation to maghemite, as already observed by Campo et al.^21^

**Figure 8:**
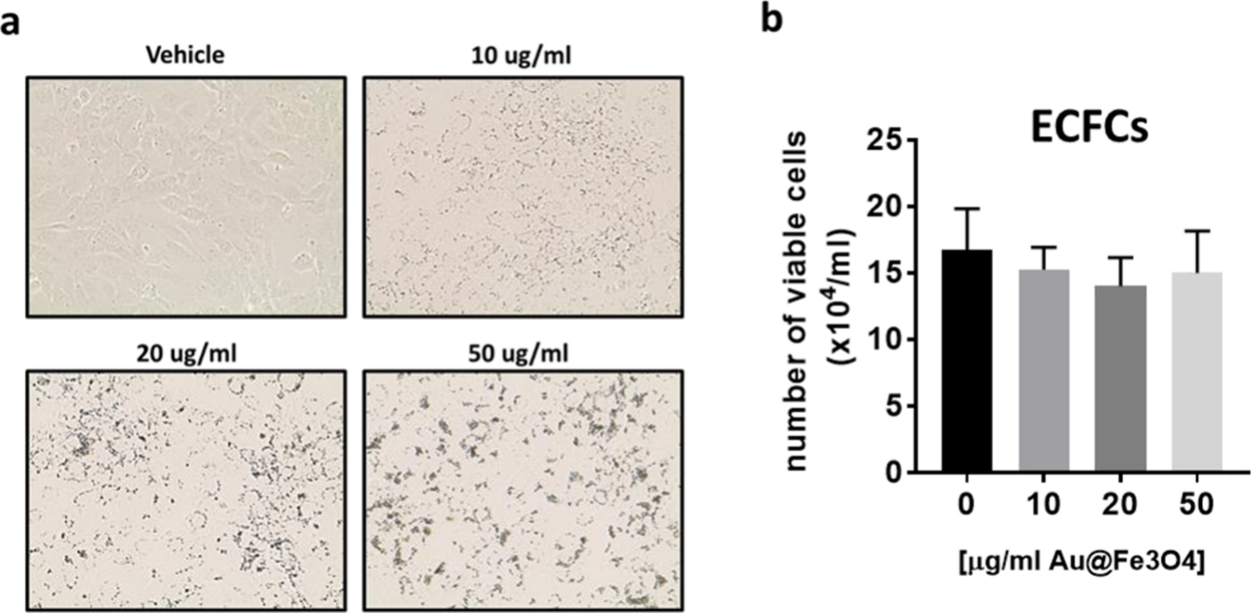
(a) Representative bright field optical images of ECFCs treated with increasing dose of Au@Fe_3_O_4_ nanostars. (b) Cell viability assessed by trypan blue staining.

**Figure 9:**
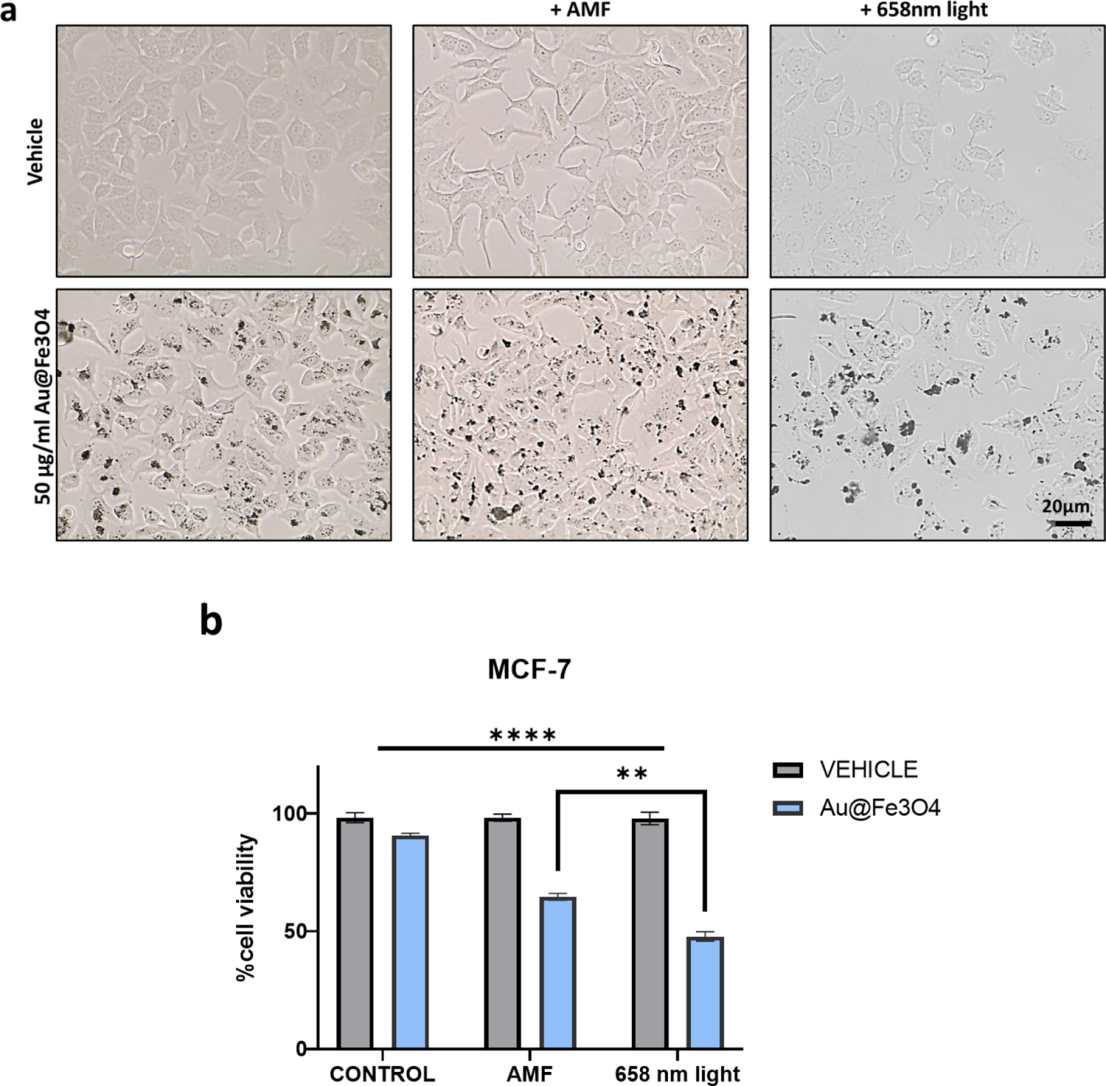
(a) Representative bright field optical images of unloaded (upper images) and loaded (50 mg/mL) MCF7 tumor cells before and after exposition to the AMF or to 658 nm light. (b) Cell viability by trypan blue staining. **p < 0.01 indicates significant difference between Au@Fe_3_O_4_ nanostars loaded MCF7 exposed to AMF or 658 nm light; ****p < 0.0001 indicates significant difference between Au@Fe_3_O_4_ nanostar-loaded MCF7 before and after AMF or light treatment.

Before assessing the biomedical potential of Au@Fe_3_O_4_ nanostars, a biocompatibility study was performed on ECFCs well known for their capacity to differentiate into mature endothelial cells which line all blood vessels. Thus, ECFCs represent an essential test bench for any in vivo circulating nanodevices. Exposure of ECFCs to increasing Au@Fe_3_O_4_ nanostar concentration (10, 20, and 50 μg/mL) for 24 h resulted in a dose-dependent cellular uptake, as observed with a conventional optical microscope under white light exposure. Figure 8 undoubtedly shows the internalized nanostars loads into ECFCs, identifiable as the black areas inside the cells. As reported in Figure 9, we observed no statistically significant changes in the number of viable cells compared to control cells even at high concentration (up to 50 μg/mL), confirming the negligible toxicity and excellent biocompatibility of this nanodevice.

Then, to evaluate the magneto-mechanical stress and the photothermal effects on cancer cells, we carried out in vitro cytotoxicity tests on unloaded and loaded MCF7 tumor cells before and after AMF application or 658 nm light exposure (Figure 9). Cells treated, as described above, with the highest dose (50 μg/mL) of Au@Fe_3_O_4_ for 24 h were able to uptake significant amount of metal nanostars, as shown by the bright field microscopy images in Figure 9a. After the nanostar incorporation, cells were then exposed to the AMF or light, as described in the Experimental Section. In both cases, the cell viability was significantly reduced to 65% after AMF stimulation or to 45% after light exposure (Figure 9b). The efficacy of the thermotransductive and magneto-mechanical potential of Au@Fe_3_O_4_ was also confirmed by morphological changes including cell shrinkage after AMF stimulation or light exposure (Figure 9a).

Recently, there has been an increased interest in iron–gold-based hybrid nanostructures, due to their combined outstanding optical and magnetic properties resulting from the usage of two separate materials. For instance, nanohybrids combining magnetic and gold NPs have been mainly employed for gene and drug delivery applications.^72−76^ Instead, few works have described the synthesis of Au@iron oxide NPs with considerable energy-to-heat conversion under both AMFs (Δ*T* ≈ 2.5 °C) and NIR light (Δ*T* ≈ 17 °C).^77^ However, even though the authors showed the biocompatibility of these hybrid platforms, they do not prove the efficacy of both the magnetic and plasmonic components in providing an antitumoral activity. On these bases, the Au@Fe_3_O_4_ nanostars described in this study represent a step forward in the synthesis and development of hybrid nanostructures with a promising biomedical potential since they are easily taken up by cancer cells and exhibit very low cytotoxicity and great efficacy in vitro. Moreover, we wish to stress that typical laser powers used in photothermal treatments are generally within the range 2–20 W/cm^2^, which is at least 1-order of magnitude higher than the one employed in our work, which makes our results very interesting in view of real applications.^53,78,79^

## Conclusions

For the first time, we have successfully synthesized through a seeded-growth approach Au@Fe_3_O_4_ MP-NPs with an extremely uniform nanostar morphology consisting of a 20 nm Au core and a 20 nm thick magnetite shell, which is able to red shift the plasmonic resonance at 640 nm. The detailed electron microscopy analysis highlighted the multifaceted nature of the Au core, which induces the epitaxial growth of a high crystalline and homogeneous iron oxide shell, conferring unusual star-like morphology to the final nano-heterostructure. Thanks to the high spin order of the ferrite shell, the nanostars exhibit a high magnetic moment which is a fundamental requirement for application in biomedicine. The purity of the magnetite phase was also assessed through MCD, which confirmed the absence of oxidized products which may form under air exposure, while multi-wavelength hysteresis loops collected through MCD confirmed the homogeneity of the magnetic phase with no contribution from the Au core. Optical extinction spectroscopy measurements revealed a plasmonic resonance at 640 nm, within the so-called first therapeutic window, coming from the Au core of the heterostructure which is strongly red-shifted compared to pure Au NPs, in agreement with analytical calculations based on Mie theory. Moreover, with respect to other Au@Fe_3_O_4_ CS NPs reported in the literature, we reached the maximum red shift of the plasmonic resonance achievable in Au@Fe_3_O_4_ CS systems due to a complete, homogeneous, and thick magnetite shell. The optical response of nanostars can be leveraged for photothermal therapy, as demonstrated by experiments performed by irradiating water stable suspension and cancer cell suspensions. All these features along with the effectiveness of the magneto-mechanical treatment make the nanostars a promising tool to be exploited in technological and biomedical applications, such as the magneto-photothermal treatments.

## Supporting Information Available

Tomography model of Au^(0)^ seeds and Au@Fe_3_O_4_ nanostars; temperature-dependent magnetic properties; optical model to simulate the plasmonic response; consideration about the optical losses of the shell; UV–vis spectra of Au@Fe_3_O_4_ in a polystyrene film; simulation of the expected MCD contribution from Au core; discussion about MCD transition of magnetite; and photothermal characterization (PDF)
