# Supplementary material for: Star-Shaped Magnetic-Plasmonic Au@Fe3O4 Nano-Heterostructures for Photothermal Therapy
Source: ACS Appl Mater Interfaces. 2022 Jun 16;14(25):29087–98. doi: 10.1021/acsami.2c04865 (PMC9247976; doi:10.1021/acsami.2c04865)
Supplement: Supplementary file 1 — am2c04865_si_001.pdf [file am2c04865_si_001.pdf]

Supporting Information

Star shaped magnetic-plasmonic $Au@Fe_3O_4$ nano-heterostructures for photothermal therapy

Beatrice Muzzi,[$,◊,1] *Martin Albino,*[$,&,1] *Alessio Gabbani,*[#] *Alexander Omelyanchik,*[§] *Elena Kozenkova,*[§] *Michele Petrecca,*[&] *Claudia Innocenti,*[$] *Elena Balica,*[&] *Alessandro Lavacchi,*[$] *Francesca Scavone,*[⁑] *Cecilia Anceschi,*[⁑] *Gaia Petrucci,*[#] *Alfonso Ibarra,*[▽] *Anna Laurenzana,*[⁑] *Francesco Pineider,*[#] *Valeria Rodionova,*[§] *Claudio Sangregorio*[$,&*]

[$] Institute of Chemistry of Organometallic Compounds – C.N.R. 50019 Sesto Fiorentino (FI), Italy

[◊]Dept. of Biotechnology, Chemistry and Pharmacy, University of Siena 1240, I-53100 Siena, Italy

[&]Department of Chemistry 'Ugo Schiff' & INSTM, University of Florence, 50019 Sesto Fiorentino (FI), Italy.

[§]Institute of Physics, Mathematics and Information Technology, Immanuel Kant Baltic Federal University, 236008 Kaliningrad, Russia

[#]Department of Chemistry and Industrial Chemistry & INSTM, University of Pisa, 56126 Pisa, Italy.

[▽]Laboratorio de Microscopias Avanzadas (LMA), Universidad de Zaragoza, 50018- Zaragoza, Spain

[⁑] Department of Experimental and Clinical Biomedical Sciences, University of Florence, 50134 Firenze, Italy

[1] Equally contributing authors.

Keywords: core@shell, heterostructures, Au@Fe_3O_4, nanostar, magnetic-plasmonic, photothermal-therapy.

1. Tomography

Au Mg_Wmediaplayer.mpeg

Video: Tomography model of $Au^{(0)}$ seed and Au@Fe_3O_4 nanostars obtained by HRTEM analysis.

2. Temperature dependent magnetic properties

Figure S1a shows the temperature dependence of the coercive field. The experimental values are well described by the Kneller's law:

$$H_C = H_C(0)\left[1 - \frac{T}{T_B}\right]^{\beta} \qquad (1)$$

where $H_C(0)$ is the coercive field at 0 Kelvin, T_B is the blocking temperature and the exponential factor β is 0.5 or 0.77 for oriented or randomly oriented assembly of non-interacting nanoparticles, respectively.[S1,S2] However, a change in the slope was observed in correspondence to the Verwey transition temperature. The best fit parameters obtained are $\mu_0 H_C(0)$ = 75.9 mT, T_B = 548 K and β = 0.30. The β factor is smaller than that theoretically predicted. A similar deviation is often observed in real system due to the change of the intrinsic magnetic anisotropy upon varying temperature.[S3,S4]

The temperature dependence of the saturation magnetization is satisfactorily fitted to the Bloch's law[S5]

$$M_S = M_S(0)(1-(T/T_C)^{\alpha}) \qquad (2)$$

with $M_S(0)$ = 82.7 (Am^2/kg), T_C = 922 K, and α = 2.29. The obtained Curie Temperature, T_C is slightly higher than the expected one (858 K for a bulk magnetite[S6]).

The effective cubic magnetic anisotropy of our nanoparticles can estimated as $K_{eff} = H_C(0)M_S(0)/0.64 = 5.14\ 10^4\ J/m^3$.

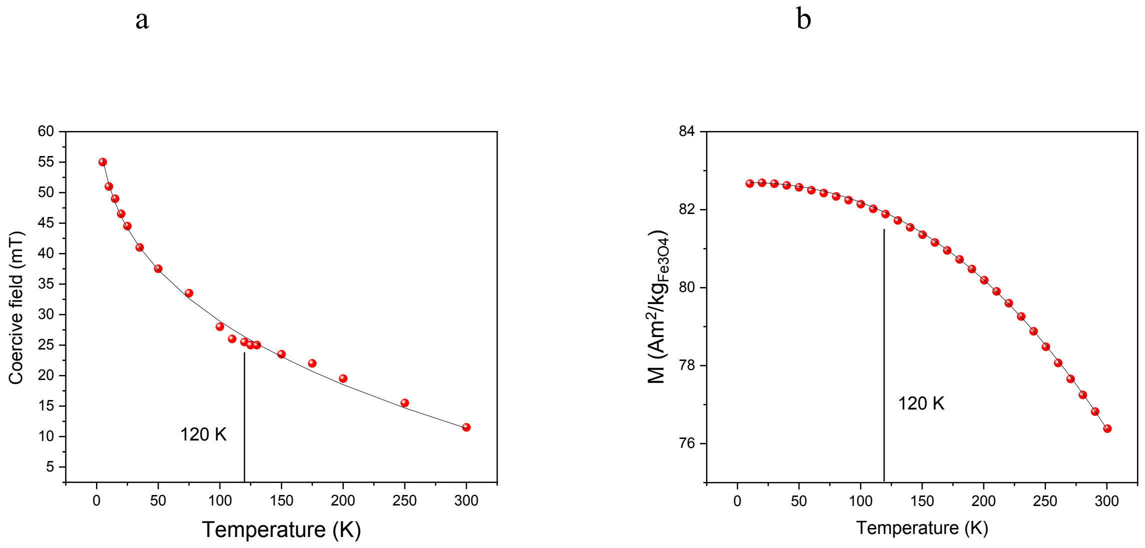

Figure S1. *a*) Evolution of experimental H_C values (red dots) as a function of the temperature and best fitting curve (black line) to the Kneller's law ($\mu_0H_C(0) = 75.9$ mT, $T_B = 548$ K and $\beta = 0.30$); *b*) M_S (red dots) decay with increasing the temperature and best fit curve (black line) to the Bloch law, $M_S = M_S(0)(1-(T/T_C)^{\alpha})$, ($M_S(0) = 82.7$ (Am^2/kg), $T_C = 922$ K, $\alpha = 2.29$).

3. Optical properties

3.1 Optical model to simulate the plasmonic response.

To simulate the plasmonic response of the Au@Fe_3O_4 NPs we employed the dipolar quasi-static polarizability of a core@shell system, within the assumption of particles much smaller than incoming wavelengths (R<<l):[S7]

$$\alpha(\lambda) = \frac{4}{3}\pi(R+d)^3 \frac{[\varepsilon_s(\lambda) - \varepsilon_m][\varepsilon_c(\lambda) + 2\varepsilon_s(\lambda)] + (\frac{R}{R+d})^3[(\varepsilon_c(\lambda) - \varepsilon_s(\lambda))][(\varepsilon_m + 2\varepsilon_s(\lambda))]}{[\varepsilon_s(\lambda) + 2\varepsilon_m][\varepsilon_c(\lambda) + 2\varepsilon_s(\lambda)] + (\frac{R}{R+d})^3[(\varepsilon_c(\lambda) - \varepsilon_s(\lambda))][2\varepsilon_s(\lambda) - 2\varepsilon_m]} \quad (3)$$

where R is the core radius, d is the shell thickness, e_m is the dielectric constant of the medium (in our case the solvent), e_s and e_c are the dielectric functions of the shell and core respectively, taken from

experimentally determined values reported in the literature.[S8,S9] Size correction of the dielectric function of the gold core is introduced according to Kreibig and Vollmer.[S10]

The extinction cross section is then calculated from the imaginary part of the polarizability, according to the following equation:

$$\sigma(\lambda) = k\sqrt{\varepsilon_m} Im[\alpha(\lambda)] \qquad (4)$$

The calculations were performed for different shell thicknesses, ranging from 0 to 60 nm, with a fixed core radius of 10 nm.

The calculations for shell thickness below 30 nm are reported in the main text (Figure 4), while the one for shells larger than 30 nm is reported below.

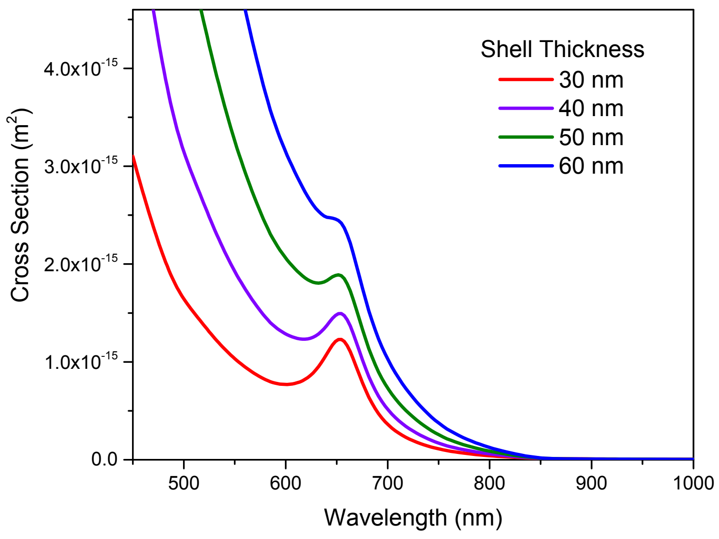

Figure S2: Cross section of core@shell Au@Fe_3O_4 NPs for thickness in the range 30-60 nm.

3.2 Consideration about the optical losses of the shell.

The increase in extinction cross section in the core@shell NPs as a function of the shell thickness can be ascribed to two reasons: i) optical transitions due to the shell become more significant as they increase with the shell volume; ii) the increase in the local refractive index around the Au core also enhances the extinction cross section of the core, due to an electromagnetic effect.

To evaluate the shell contribution as a function of its thickness, we add here the analytical simulation of the contribution of the bare shell (neglecting the one from the core, which is here considered with the same refractive index of the solvent) compared to the full core@shell system (Figure S3) for four

shell thickness: 5, 10, 20 and 30 nm. The contribution of the shell at the plasmonic peak wavelength with respect to the total extinction cross section is 3.4%, 6.5%, 14% and 28%, respectively for shell thickness of 5, 10, 20, 30 nm. The simulations indicate that the increase in extinction cross-section at the plasmonic resonance for the core@shell system with thickness above 20 nm is mainly due to the shell contribution, which scales with the shell volume. Indeed, if we subtract the calculated shell contribution from the cross section of the core shell, we obtain similar extinction cross section at the peak maximum for the case of 20 nm and 30 nm shell.

To assess which is the optimal shell volume two issues must be taken into account: First, shifting the peak toward longer wavelengths is desired, which is already maximized at 20 nm of shell thickness; Secondly, the increase in cross-section is also desired, provided that the shell contribution is not too high to decrease the light that reaches the core. In light of these considerations, a 20 nm thickness appears as the best compromise. Indeed, further increasing the shell thickness above 20 nm is not a great advantage as it shifts only slightly the plasmonic peak, and it does not increase the Au core absorption but only the one of the magnetite shell.

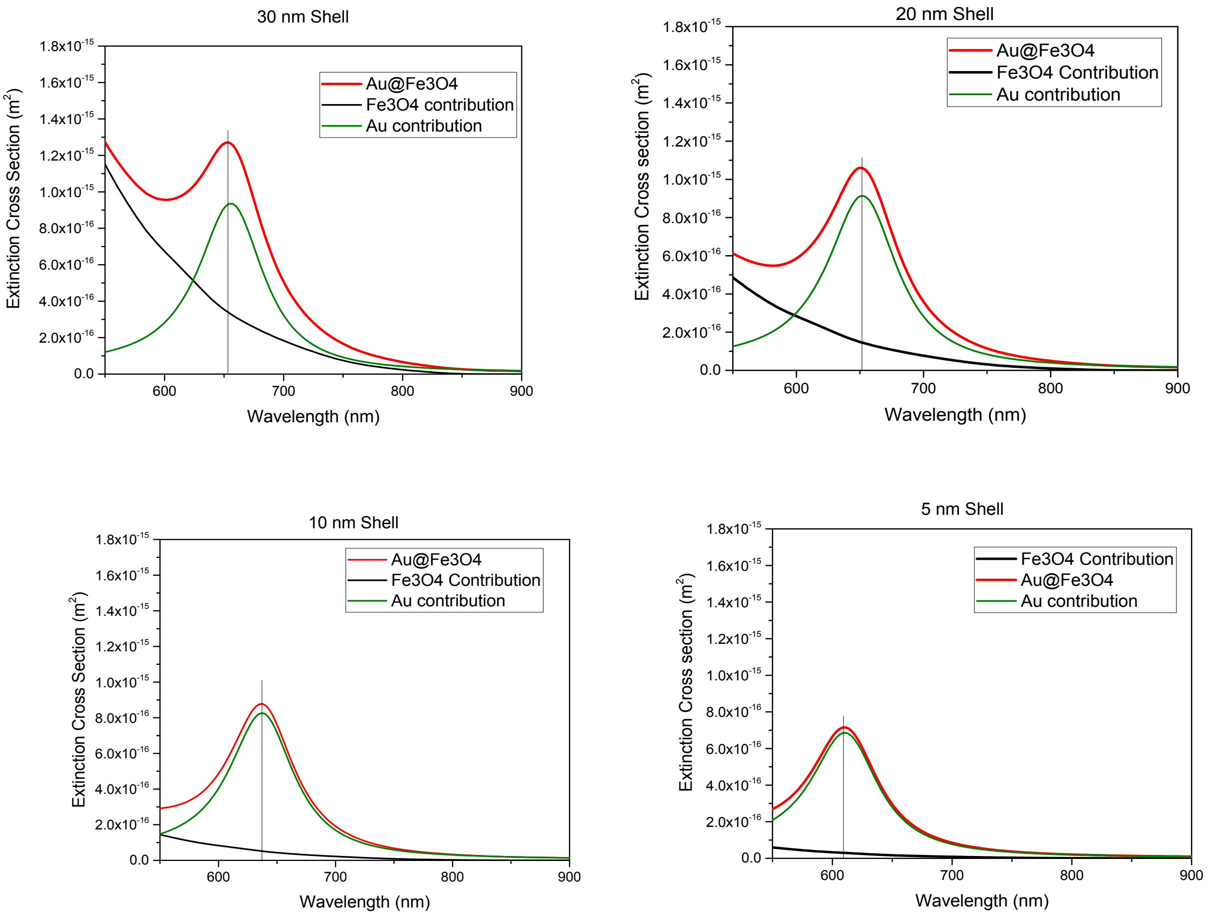

Figure S3: Analytical calculations of the extinction cross section for a Au@Fe_3O_4 core@shell system with 20 nm of Au core diameter and shell of various thickness (30, 20, 10 and 5 nm). The cross section of the bare shell was calculated using the same analytical model described in the text but setting the core refractive index equal to the one of the solvent. The contribution of the core was obtained subtracting the shell contribution from the total extinction cross section of the core@shell NP.

3.3 Comparison of the UV-vis spectra of the Au@Fe_3O_4 NPs dispersed in hexane and in a polystyrene film.

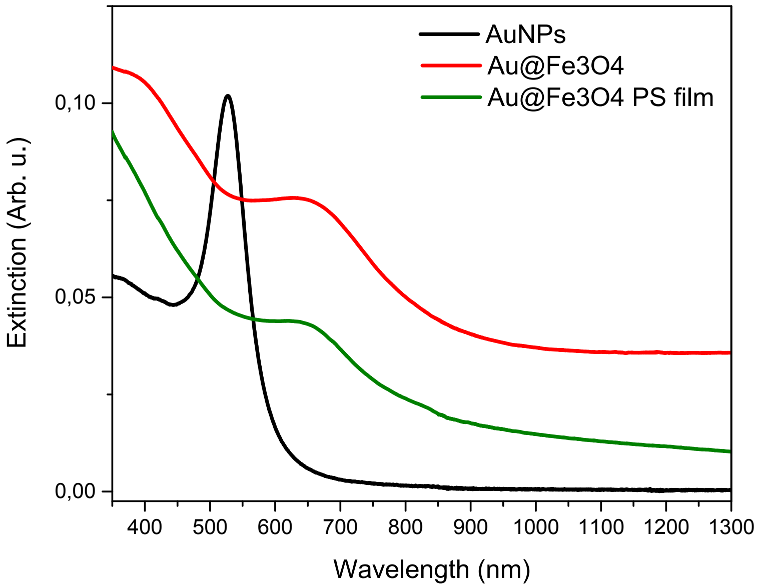

Figure S4: UV-vis-NIR extinction spectra of the Au seeds (black line), of the Au@Fe3O4 heterostructures in hexane dispersion (red line) and in the PS film (green line).

4. MCD: Simulation of the expected MCD contribution from the Au core

In the first approximation, the MCD contribution due to the LSPR can be simulated starting from the optical peak and assuming a rigid cyclotron shift of the plasmonic resonance. To this aim, the extinction spectrum was fitted, extracting the parameters describing the LSPR contribution, namely peak width, peak area and peak position in energy.

For the fitting, we used a sum of peak functions, which take into account all the optical transitions occurring in the sample (Figure S4 a). The high energy peak component is ascribed to the interband transitions, while the one at one to the LSPR of the Au core. A third peak is necessary to completely reproduce the sample spectrum, which may arise from slight aggregation of the particles during the formation of the polymer film. The peak parameters of the Lorentz function (*equation 5*) used for the LSPR were extracted: E_0 = 1.88 eV (l_0=660 nm) is the LSP resonance energy (wavelength); C = 0.147 peak amplitude; γ = 0.781 eV is the peak width.

$$A = \frac{2C}{\pi}\frac{\gamma}{4(E-E_0)^2+\gamma^2} \qquad (5)$$

Using the same peak parameters of the LSPR component, the MCD contribution of the Au core of the heterostructure was simulated, within the assumption that no interaction is occurring between the plasmonic and the magnetic part, by calculating the difference between two peak functions having the same parameters of the LSPR extinction peak but oppositely shifted in energy by a factor $\pm\hbar\omega_C/2$ (where ω_C is the cyclotron frequency) with respect to the extinction peak.

The extinction peak parameters are thus inserted into *Equation 6-7*:

$$\Delta A = \frac{2C}{\pi}\frac{\gamma}{4(E-E_0+\Delta E)^2+\gamma^2} - \frac{2C}{\pi}\frac{\gamma}{4(E-E_0-\Delta E)^2+\gamma^2} \qquad (6)$$

$$\Delta E = \hbar\frac{eB}{2m_e} \qquad (7)$$

where $\hbar$ is the Plank constant, e the electron charge, B is the applied magnetic field (equal to 1.4 T for our experiments) and m_e is the free electron mass.

The as-simulated MCD for our heterostructure has a magnitude in the order of 10^{-5} (Figure S4), which is two orders of magnitude weaker than the experimental signal.

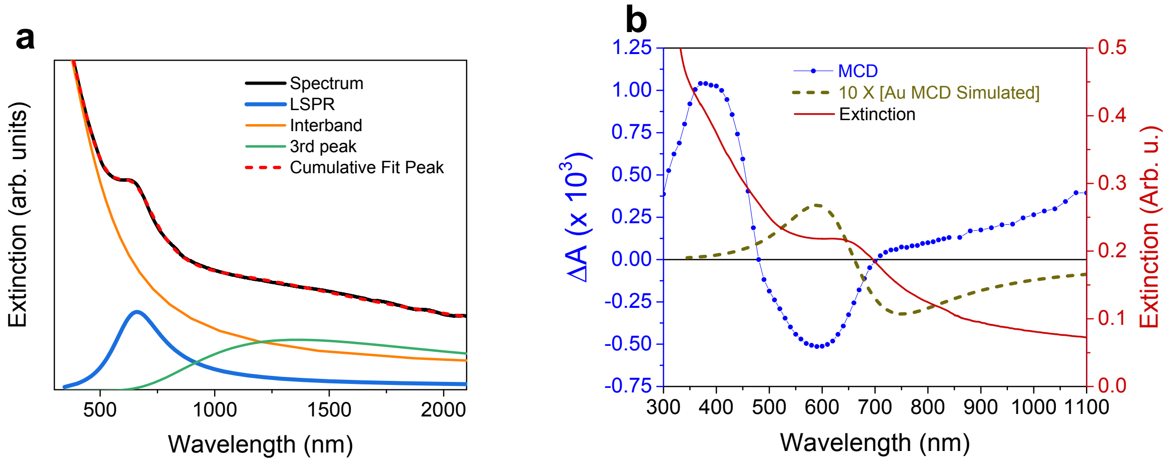

Figure S5: Fit of the extinction spectrum performed using a sum of three peak functions (a) and experimental MCD spectrum of the heterostructures compared with the simulated MCD contribution arising from the plasmonic part, which is here multiplied by a factor of 10 to make its identification in the graph easier (b).

4.1 Discussion about MCD transition of Magnetite

A discussion related to the Magneto-Optical transitions of Fe_3O_4 is provided below.

Square brackets indicate ions in octahedral coordination, while parenthesis indicates tetrahedral coordination. Charge transfer transitions involves different cations: electron transfer between neighbouring cations (IVCT) or between cations in different crystallographic sites (ISCT).

According to the work by Fontin *et al.*[S11], the following transitions are expected (their position is also indicated with vertical lines on our experimental MCD spectrum in Figure S6):

Table S1.

Number	Energy	type	Transition	MO line shape
1	3.94 eV	ISCT	$[Fe^{2+}]t_{2g} \rightarrow (Fe^{2+})t_2$	- diamagnetic
2	3.93 eV	IVSCT	$(Fe^{3+})t_2 \rightarrow [Fe^{2+}]e_g$	+ diamagnetic
3	3.46 eV	ISCT	$[Fe^{3+}]e_g \rightarrow (Fe^{2+})t_2$	+diamagnetic
4	3.11 eV	IVCT	$[Fe^{2+}]t_{2g} \rightarrow (Fe^{2+})e$	+diamagnetic
5	2.61 eV	ISCT	$(Fe^{3+})t_2 \rightarrow [Fe^{2+}]t_{2g}$	-diamagnetic
6	1.94 eV	IVCT	$[Fe^{2+}]t_{2g} \rightarrow [Fe^{2+}]e_g$	+ paramagnetic
7	0.56 eV	IVCT	$[Fe^{2+}]t_{2g} \rightarrow [Fe^{2+}]t_{2g}$	- paramagnetic

In our spectrum, a broad positive signal in the range 300-500 nm includes the first 4 transitions reported in the table. Due to the opposite diamagnetic terms (two opposite S-shaped signals results in an apparent peak-like signal), the convolution of these transition results in a positive peak centered around 3.25 eV, with a shoulder at 3.9 eV. At 2.6 eV, the MCD signal crosses the zero. The transition $(Fe^{3+})t_2 \rightarrow [Fe^{2+}]t_{2g}$ is expected at this energy, and has a diamagnetic line shape of opposite sign with respect to the transition at 3.11 eV. This transition is thus identified as a derivative-like signal centered at 2.6 eV, which introduces a low energy shoulder in the broad positive peak at high energy. The negative peak around 2 eV can be assigned to the transition $[Fe^{2+}]t_{2g} \rightarrow [Fe^{2+}]e_g$, as it is close to the value reported by Fontin et al. (1.94 eV) and it appears as a peak, typical of the paramagnetic line

shape. The transition $[Fe^{2+}]t_{2g} \rightarrow [Fe^{2+}]t_{2g}$ in our case is found at higher energy (0.85 eV instead of 0.56 eV as reported by Fontin *et al.*).

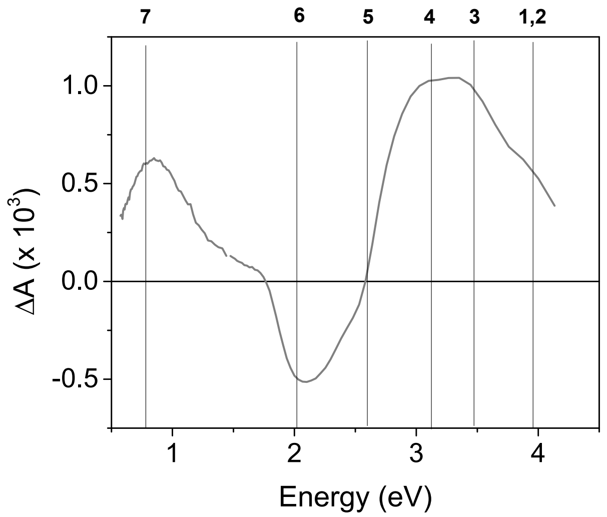

"

Figure S6: Magnetic Circular Dichroism spectrum of $Au@Fe_3O_4$ NPs. The main transitions reported in Table S1 are indicated with vertical lines. The numbers on the top correspond to the transitions indicated in Table S1.

5. Photothermal Characterization

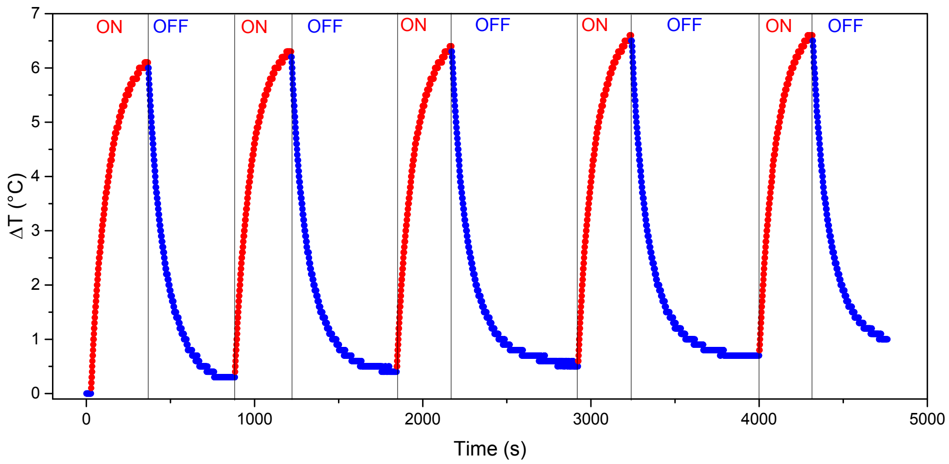

Figure S7. Cycles of time dependent temperature build-up in water dispersion of PEI coated $Au@Fe_3O_4$ nanostars, irradiated by a laser diode at 658 nm (red curve, laser power 300 mW/cm^2). Data points after switching off the light source are marked in blue.

6. References

[S1] E.F. Kneller, F.E. Luborsky J. Appl. Phys., 34 (1963), 656.

[S2] X. Batlle, M. Garcia del Muro, J. Tejada, H. Pfeiffer, P. Goand, E. Sinn J. Appl. Phys., 74 (1993), 3333.

[S3] C. Nayek, K. Manna, G. Bhattacharjee, P. Murugavel, I. Obaida Magnetochemistry 3 (2017), 19.

[S4] G. Gomide, R. Cabreira Gomes, M. Gomes Viana, A. Fabiano Cortez Campos, R. Aquino, A. López-Ortega, R. Perzynski, J. Depeyrot J. Phys. Chem. C 126 (2022), 3.

[S5] Bloch, F. Zur theorie des ferromagnetismus. Z. Phys. 61 (1931), 206.

[S6] Néel; Louis. Propriétés Magnétiques Des Ferrites ; Ferrimagnétisme et Antiferromagnétisme. *Ann. Phys. (Paris).* **1948**, *12*, 137–198

[S7] Absorption and Scattering of Light by Small Particles; Bohren, C. F.; Huffman, D. R., Eds.; Wiley-VCH Verlag GmbH: Weinheim, Germany, 1998. DOI: 10.1002/9783527618156

[S8] Battie, Y.; Stchakovsky, M.; Neveu, S.; Jamon, D.; Garcia-Caurel, E. Synthesis and Study of γ-Fe2O3 and CoFe2O4 Based Ferrofluids by Means of Spectroscopic Mueller Matrix Ellipsometry. J. Vac. Sci. Technol., B: Microelectron. Process. Phenom. 2019, 37, 062929 DOI: 10.1116/1.5121286;

[S9] Johnson, P. B.; Christy, R.-W. Optical Constants of the Noble Metals. Phys. Rev. B 1972, 6, 4370, DOI: 10.1103/PhysRevB.6.4370

[S10] Kreibig, U.; Vollmer, M. Optical Properties of Metal Clusters; Springer-Verlag: Berlin, 1995.

[S11] Fontijn, W. F. J.; Van Der Zaag, P. J.; Feiner, L. F.; Metselaar, R.; Devillers, M. A. C. A Consistent Interpretation of the Magneto-Optical Spectra of Spinel Type Ferrites (Invited). J. Appl. Phys. 1999, 85 (8 II A), 5100–5105.
